# Analysis of the Possibility of Using Selected Tools and Algorithms in the Classification and Recognition of Type of Microstructure

**DOI:** 10.3390/ma16216837

**Published:** 2023-10-24

**Authors:** Michał Szatkowski, Dorota Wilk-Kołodziejczyk, Krzysztof Jaśkowiec, Marcin Małysza, Adam Bitka, Mirosław Głowacki

**Affiliations:** 1 Faculty of Metals Engineering and Industrial Computer Science, AGH University of Science and Technology in Krakow, al. Mickiewicza 30, 30-059 Kraków, Poland; mszatkowski97@gmail.com (M.S.); kjakow@agh.edu.pl (K.J.); marcin.malysza@kit.lukasiewicz.gov.pl (M.M.); adam.bitka@kit.lukasiewicz.gov.pl (A.B.); glowacki@metal.agh.edu.pl (M.G.); 2 Łukasiewicz Research Network—Krakow Institute of Technology, Zakopiańska 73, 30-418 Kraków, Poland; 3 Faculty of Natural Sciences, Jan Kochanowski University of Kielce, ul. Żeromskiego, 25-369 Kielce, Poland

**Keywords:** classification of microstructures, cast iron, quality assessment

## Abstract

The aim of this research was to develop a solution based on existing methods and tools that would allow the automatic classification of selected images of cast iron microstructures. As part of the work, solutions based on artificial intelligence were tested and modified. Their task is to assign a specific class in the analyzed microstructure images. In the analyzed set, the examined samples appear in various zoom levels, photo sizes and colors. As is known, the components of the microstructure are different. In the examined photo, there does not have to be only one type of precipitate in each photo that indicates the correct microstructure of the same type of alloy, different shapes may appear in different amounts. This article also addresses the issue of data preparation. In order to isolate one type of structure element, the possibilities of using methods such as HOG (histogram of oriented gradients) and thresholding (the image was transformed into black objects on a white background) were checked. In order to avoid the slow preparation of training data, our solution was proposed to facilitate the labeling of data for training. The HOG algorithm combined with SVM and random forest were used for the classification process. In order to compare the effectiveness of the operation, the Faster R-CNN and Mask R-CNN algorithms were also used. The results obtained from the classifiers were compared to the microstructure assessment performed by experts.

## 1. Introduction

The problem addressed in this research described in this article concerns the automatic classification of the correctness of cast iron based on images of the microstructure. In the examined case, work was carried out on the example of cast iron microstructures, where an important aspect of the correctness of the casting is the shape of the graphite. The solution described herein focuses on the recognition of this shape using selected methods and the subsequent classification of images in terms of the correctness of the obtained alloy.

The problem of classifying Images of microstructures is the subject of many studies described and presented in the subsequent literature [1,2,3,4]. These works highlight the tools and techniques used in recent years. Their analysis allows us to determine the direction of development of tools to solve this problem. These works are of a review nature [1,2] or focus on a specific problem defined in materials engineering [3] or a comparison of various methods used in the classification problem [4,5,6,7,8,9,10,11]. When solving this problem, it is also worth thoroughly analyzing the principles of operation of tools and algorithms based on artificial intelligence [12,13,14,15,16,17,18].

Based on the identified problem to be solved and the analysis of the literature indicated above, the purpose and scope of the described research were developed in relation to the analysis of the capabilities of the tools and algorithms used to solve a specific task. The conclusion that comes to mind after analyzing all the cited articles is the need to test the selected algorithms on a data set dedicated to use in this work. Many authors have already developed methods that classify microstructures using machine learning but their effectiveness in relation to cast iron microstructures is not satisfactory to be used in serial solutions; therefore, work in this area is still justified.

## 2. Materials and Methods

**Figure 1:**
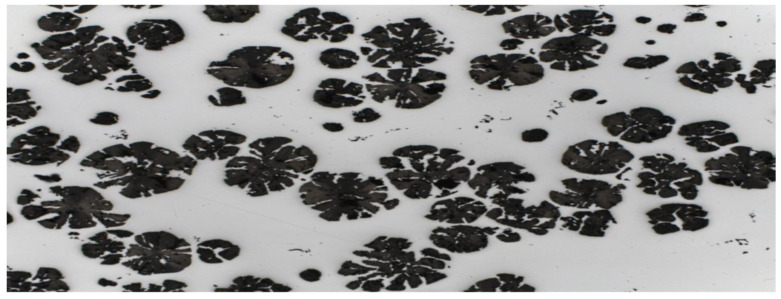
Examples of images of cast iron microstructures used in the research.

**Figure 2:**
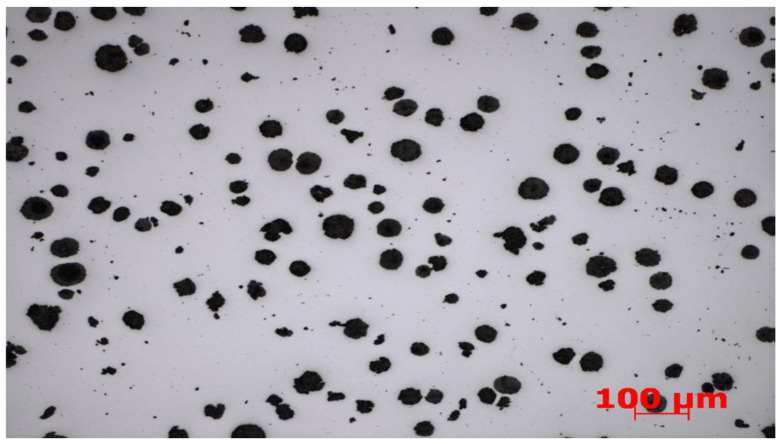
Examples of images of cast iron microstructures used in the research.

The first stage of work on the received set was to find an appropriate method that would allow many objects to be automatically marked at once in order to assign them to a specific class. One of the first things to be noticed is the characteristics of the analyzed photos. Sample data are shown in Figure 1 and Figure 2.

In order to recognize and classify selected species of microstructures, the possibility of using two types of convolution-al networks and two classical methods was examined. The first is a standard convolutional network used for object detection—in this case, it was decided to use the effective RetinaNet architecture. Convolutional neural network architectures, as summarized in [1], are a family of special neural networks adapted for image operations. They are able to perform a number of tasks such as basic photo classification, detection of objects in the photo and their classification or image segmentation and classification of individual sections. Networks of this type are characterized by the use of filters that allow the formation of maps of features, each of which relates to some pattern in the image. The mentioned limitation of parameters is performed with the help of pooling layers, which are also responsible for reducing the dimensions of a given layer. The second is a network that uses image segmentation to perform semantic segmentation. In the case of the classical approach, there will be two solutions. The first one is a part of the HOG algorithm and the second is part of the decision tree and the SVM classifier supported by the sliding window method, respectively. HOG (histogram of oriented gradients) is a solution used to map features in an image. SVM is a binary classifier that identifies a hyperplane of N-1 dimensions for a group of points in dimension N, which was described in [2]. Decision trees are presented in [3]. The national standard [19] is helpful in the analysis of shape.

### Dataset Analysis and Augmentation

The microstructure images that were used were microstructure images of cast iron microstructures (with different types of graphite) with different approximations or sizes of microstructure. In the graphite microstructure classification standard the types of graphite were separated in terms of form. During this work, the distribution of other precipitates in the microstructure was not taken into account as the samples were not sufficient for this. Most possibilities are provided by forms I, IV and VI (according to classification from), which were definitely the most numerous in the microstructure image whereby they could be marked using the prepared tool. Therefore, it was decided that further research would focus on the detection of these three types of objects. The scale at which photos of microstructures were taken for the tested alloys is not the same. It was mostly not problematic due to the size diversity in the microstructure image, sometimes independent of the zoom. However, some microstructure images had too small an approximation to be able to conveniently obtain data from them. This would not be such a serious problem if the microstructure image had a higher resolution. The most difficult type of microstructure image in terms of working on them turned out to be those that showed objects of form I. Forms IV and VI) have, to put it simply, a uniform general shape and the division between them is visible to the naked eye. However, for form I, a situation was observed where the entire image is a kind of labyrinth and the shape of the objects allows them to intertwine with each other. Another thing to note in this case is that these objects are much more problematic in terms of their size. Very small elements in forms IV and VI are numerous but larger objects are easily distinguished from them. In the case of form I, it is a more difficult task because there are equally many elements of each size on the tested samples and the difference in their sizes is not so striking. The collection in question was quite small according to machine learning standards in terms of the microstructure images that were accessed. The fact that the convention of the solution did not allow for all the objects on them to be worked out was also of key importance. The okfinal set of microstructure images that was used in the work consisted of the majority of microstructure images showing the objects of form VI.

- Microstructure image containing objects assigned to form VI—training set—89
- Photographs containing objects assigned to form VI—test set—10
- Microstructure image containing objects assigned to form IV—training set—8
- Photographs containing objects assigned to form IV—test set—1
- Pictures containing objects assigned to form I—training set—9
- Pictures containing objects assigned to form I—training set—1

This decomposition made it necessary to create more data. The most missing objects were those assigned to form IV as they were relatively the largest in the available images and there were the fewest in one image. The collection was expanded by creating different versions of the microstructure images. Most of them were created for form IV, where two additional copies of the image were first created—one twice as small, the other twice as large—and then their versions rotated along both axes were created for each of them. For form VI, some of the microstructure images with the greatest concentration of small objects were enlarged, and later this version was cut into several smaller images. In the case of form I, all images were given a larger copy and then all were divided into smaller parts. The final set consisted of 1132 images for the training set and 150 images for the test set.

## 3. Results

### 3.1. Detection and Classification of Objects

As part of the classical methods, it was decided to analyze the behavior of the detector based on a simple scheme. The technique of a sliding window and Gaussian pyramid was used as the mechanism for checking the entire image, which allowed a small window to be moved around the entire image at different scales and capture many possible objects. Each time the window was moved, the HOG algorithm was applied to obtain a feature map of a given image element, which was then passed to the classifier, which was either an SVM or a random forest. Finally, the detected detections were filtered and the best of them was selected for one site, which could be achieved using the nonmaximum suppression method available in the imutils library. The vast majority of these detectors are solutions provided by the scikit-image library. Using this solution, only the classifier was trained. The process itself was very fast but many problems and necessary modifications to the dataset were experienced during the tests of the detector. The first problem appeared at the very beginning. The detector, using SVM, had to have a specific, constant input data size. The cut objects in the data set had different shapes and sizes but for the purposes of training the network, each was given the same size, transforming the appropriate function. This was a necessary action, which, as already mentioned, disturbed the natural shape of the objects, affecting their quality as training data. However, in the case of forms IV and VI, it was not as problematic as in the case of form I. Forms IV and VI have a similar shape to a large extent, especially form VI, and, after changing their size, they retain most of their features. In the case of form I, the situation is more complicated as the objects assigned to this class were most often in the shape of a thin curved line that could be in any position. In this case, transforming such an object into a uniform shape, harmonizing it with the rest of the objects, resulted in a complete change in its proportions, which in turn disturbed the possibility of its detection by the detector. The second problem encountered while working with this type of solution was the high sensitivity of the classifiers. Confirming what is mentioned in [3], SVM and a decision tree, which are shallow learning methods, reacted strongly to small amounts of uncertain-looking images and learned to recognize them, which led to incorrect detections. An example of such behavior was when only half of the object was in the microstructure image of the training set. These microstructure images were taken on the basis of frames that tightly surrounded the objects touching their borders, and thus, if only half of the object was in the picture, the classifier learned to recognize such a case as a given class. This behavior was unfavorable for the work of the detector in the discussed case, as the sliding window method ran over many similar image fragments during operation and their classification as full-fledged objects resulted in numerous incorrect detections. This problem was compounded for form IV because its structure had an effect resembling numerous cracks and tears. This sometimes resulted in classification even in the very center of an object or even a group of objects that were close to each other. To solve this problem, at the very beginning, all microstructure images from the collection that contained incomplete objects or several combined with each other were eliminated. The approach of adding a small white border to each image was also tried but this method was unsuccessful and objects were not detected. This was due to the fact that the objects were often close to each other, which negated the existence of free space around them. The introduction of these changes resulted in a reduction in the training and test sets.

- Pictures containing one object of form VI—training set—973
- Microstructure image containing one object of form VI—test set—100
- Pictures containing one object of form IV—training set—494
- Microstructure image containing one object of form IV—test set—99

The training set was largely reduced, which was unavoidable because the deleted microstructure image contained only partial, sometimes less than half pieces of objects or several merged together. The last factor that had a strong influence on the detector’s performance was the insufficient set that was responsible for the background classification. Classical methods successfully classified objects from the test sets but when used on a full microstructure image, they turned out to assign object classes where they did not really exist or were located in the corner of the window. As mentioned, in order to prevent this phenomenon, the functionality of the prepared tool was used to divide the images into parts and use the results obtained in this way as a set to be classified as the absence of an object. The resulting series of smaller images were carefully reviewed as there were cases where the data thus provided looked too much like the correct targets for detection. After introducing these changes to the training set, the classifier’s results during training were high, as shown in Table 1. Table 2 shows the accuracy results for the random forest. The random forest slightly exceeds the SVM but both classifiers achieved very good results.

The best result was obtained for data consisting of microstructure images of objects of form I because they differ strongly in shape from other classes, so they are easily recognizable for the classifier. For form IV, it can be seen in both classifiers that the sensitivity is clearly lower than the rest of the results. This is confirmed by the previously described observations that form IV was definitely most often detected in places where the objects as such were not present. The second interesting result is the precision for form VI. This has also been noted in detection attempts, as some form IV objects were sometimes recognized by classifiers as form VI. As for the operation of the SVM with the detector alone, it was applied to two images from the test set. For the reasons described, it was only possible to successfully apply the classifier for objects of form IV and VI. The images contain frames in red and green, where the former means all detections and the latter are the detections that have been selected as final by nonmaximum suppression. Important parameters that had to be set for the detector itself should be mentioned here. The key data needed were the factor by which the main image is reduced, which is a parameter of the Gaussian pyramid, and the step with which the window will move around the image. As noted during the tests of this solution, the smaller the step and factor, the more accurately the detector was able to analyze the image. However, the factor could not be less than or equal to 1 because the image had to shrink. Another important thing was the size of the painting and the window. The window had to be the same size as the size of the microstructure image on which the classifier was trained, so in order to detect smaller objects, it was necessary to enlarge the image on which they were located. The other two parameters that could be experimented with were the NMS threshold, which affects the selection of results by this algorithm based on overlapping frames, and the detection score, which allows us to determine how good the detections considered by NMS should be. Feature acquisition by the HOG was continuous with 9 orientations and split into 3 × 3 cells, each of which was 8 × 8 pixels. In the described case, it is the detection of objects of form VI performed for an image with dimensions of 2776 × 2080, with the detection confidence threshold set at 2.0, the NMS threshold set at 0.3, the step of moving the window 8 pixels and the degree of image reduction 1.2. The SVM classifier coped with the detection of form VI objects, as indicated by both the finally selected detections and those rejected. Several errors can be observed in the form of marked fragments of objects, which, due to their shape, are probably not objects of form VI but their fragments remind the classifier of the learned shape.

**Figure 3:**
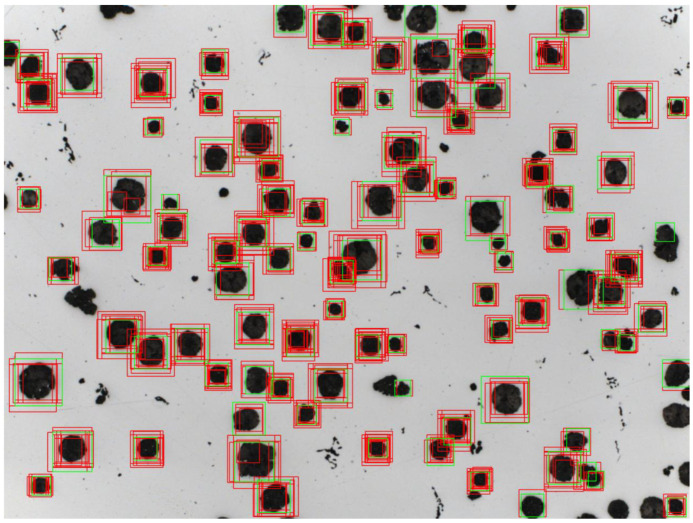
Example of operation of a classical detector for the SVM classifier.

In Figure 3, the same microstructure image shows the detection results for the random forest classifier. The images contain red and green frames, the former of which indicate all detections, and the green frames are detections that were selected as the final ones by non-maximum suppression. The black shapes are ductile iron.The same parameters as for SVM were used, with the difference that a different threshold had to be chosen for the qualitative detection threshold due to the different values adopted by the classifiers. The chosen value was 0.5. The result is very similar to what SVM presented. Differences are mainly visible in the case of a few objects, where one of the classifiers detected them, while the other missed them, but this mainly concerns the smallest ones. In addition, the random forest detected a large blank area as form VI, which is an error. After the first test, both solutions are very similar. As can be seen for form VI, the detections are correct and thanks to the appropriate modification of the set, as well as the very high accuracy with which the sliding window method was used, they correctly detect objects. The detection of form IV unfortunately did not go as well, continuing to suffer from the same problems that were reported in the observations. In terms of further results for form IV, despite numerous experiments using different parameters, better results could not be obtained. Detections were made for images with the size of 1338 × 1080 to check how much the size influenced the unsatisfactory result. The differences between the SVM and random forest are much more than in the previous test. The random forest outperformed SVM here, detecting more complete objects in the image. It should be noted, as already mentioned in the observations, that the SVM shows a greater tendency than its competitor to detect small parts of whole objects. The results for random forest have many similarities, detecting mostly the same objects; however, for SVM, this is not the case. SVM for a larger image, despite detecting a few more objects than for a smaller one, also performs a lot of incorrect classifications, creating numerous frames marking only a piece of the correct object. For further testing, smaller images were used for the two classifiers as they performed better for form IV objects. Subsequent tests that were performed took into account the changed threshold parameters for NMS and detection certainty. It was not possible to significantly improve the results, but by reducing the classifier confidence threshold to 0.45 for random forest and 1.5 for SVM and reducing the NMS threshold to 0.1, you can look at more rejected detections and see which places the algorithm most often classified as an object as a form IV object. The random forest performed better than SVM in detecting form IV objects. The first classifier, despite the frequent omission of objects or classifying their pairs as wholes, marked whole objects and detected more of them than the second classifier. The SVM almost completely could not cope with the task, marking mostly fragments of objects or their groupings as a single whole. It can be seen that although the SVM marked the right objects in places, it indicated the wrong places in most cases. Cases such as two divisions in the mentioned place, which have been eliminated by NMS in favor of a larger frame, are less frequent than the marking of pieces of objects or their entire clusters. In summary, during the described experiments, the random forest was a more effective classifier for selected images than the SVM. Recent observations are for detection time. During the experiments in the presented pictures, one could feel a big difference in the time that the two algorithms needed to perform numerous detections. Therefore, the time needed for both solutions to classify 100 microstructure images from the test set of form VI was measured. Roughly speaking, SVM completed the task in an average of 0.000009892 s per image, while the random forest completed the task in 0.000180006 s. This clearly shows that random forests, despite being more effective on harvest, needed much more time, which is especially important when you need to carefully analyze the bigger picture. From the conducted experiments on classical methods, it was concluded that the methods themselves are capable of correctly learning to detect specific forms of the discussed objects. However, this is only possible if their shape is not as diverse as in the case of form I, the detection of which turned out to be impossible for this very reason. In addition, both the random forest and SVM require an appropriate, well-thought-out training set that will cover as many possible circumstances in which a given object may find itself. In the case of the present experiments, the lack of sufficient data on form IV objects became very apparent, which contributed to poorer detection. As for the identified disadvantages of these solutions in the presented task, such solutions require the inclusion of a class representing the background in the training set, which is often difficult to implement. When creating and modifying a set for the purposes of the presented tests, the problem of frequent inability to determine when a given image section should be classified as a background, and sometimes as an object, has been noticed many times. An example would be a situation where an object that is entirely in the cutout is in the corner, and the rest represents the background. It would seem that it should be classified as a “background” element because it is not advisable for such a section to be defined in its entirety as an object during detection. However, as it turned out during the experiments, placing such a case as a background negatively affected the detection of the right objects. Thus, by not specifying contours or frames in the image, we rely much more on the interpretation of the classifier, which, as mentioned, showed high sensitivity to training data and required as many scenarios as possible in which the object could be found. This type of detector may also be inflexible, as depending on the shape of the objects you are looking for, you may need to set other parameters to get good results.

#### 3.1.1. Faster R-CNN

**Figure 4:**
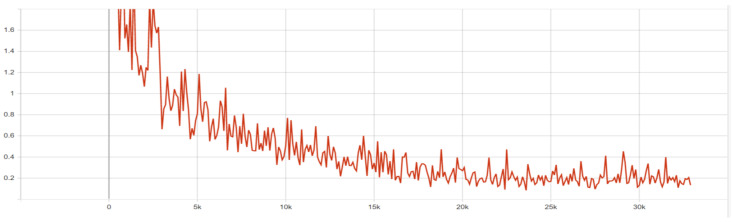
Loss function graph for overall loss.

The version of the algorithm with the smallest version of the ResNet network was chosen due to the impossibility of training another of the algorithms in this environment, which required the selection of the lightest version possible. The configuration file was modified so that the size of the batch of microstructure images that was used consisted of only one microstructure image because more of them resulted in a high computational load. Given the second deep learning algorithm in training, this choice served up all comparisons. The remaining part of the configuration file was left with default settings because, during network training, there were no problems related to its stability. The configuration file for Faster R-CNN was divided into several parts. These were: configuration of the model itself, configuration of training, configuration of evaluation and configuration of paths to annotation files. The configuration of the model consisted of the number of classes that the algorithm will accept, settings of the image resolution change mechanism, feature extractor settings, anchor frame generator settings and hyperparameters of the first-phase detector. Next were the settings for the second-phase detector and post-processing as well as all the weights and thresholds that the network needs. The network training configuration contained information such as batch size, number of steps and optimizer settings, including initial learning speed and decrease. There were also settings for the fine-tuning process. The evaluation setup mainly contained the batch size and the type of metrics used for it, which were COCO metrics. The network was trained for 32,000 steps, observing its progress with the help of the tensorboard program, which allows you to display graphs related to, for example, in this case, loss functions and average precision and sensitivity factors. Learning was stopped due to the stabilization of all loss functions. For the graph presented in Figure 4, the vertical axis means the value of the loss function in a given step, and the horizontal axis means the number of steps.

Loss functions were successively decreasing, mostly maintaining a stable level of jumps in value. The location losses of the frame classifier have the largest jumps between the lowest and highest values. The loss functions for RPN stabilized the fastest, because while for the frame classifier, similar values were maintained at around 20,000 steps, for RPN, it happened for loss of location at about 10,000 steps, and for loss of objectivity, at about 15,000 steps. Both also have an advantage in smaller value differences. It can be concluded from this that the region proposals learned more effectively than the classifier. The obtained result was also evaluated, as presented in Table 3.

**Figure 5:**
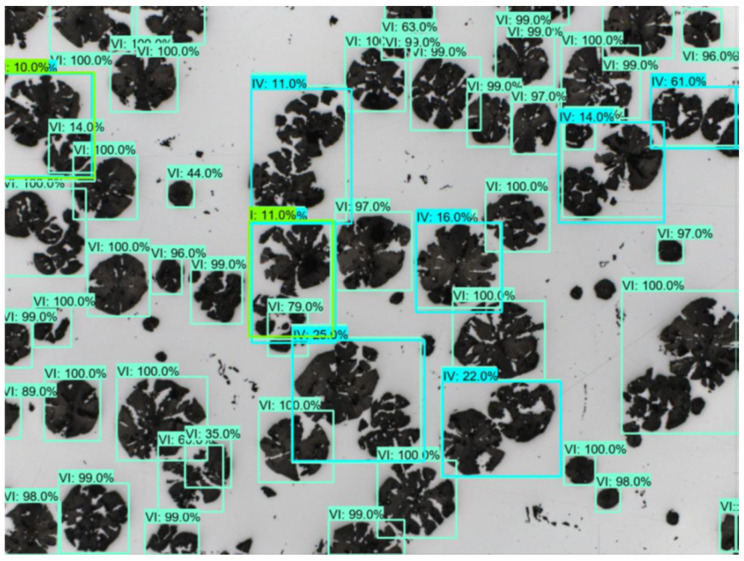
Operation of the Faster R-CNN detector on an image containing mostly form IV objects.

**Figure 6:**
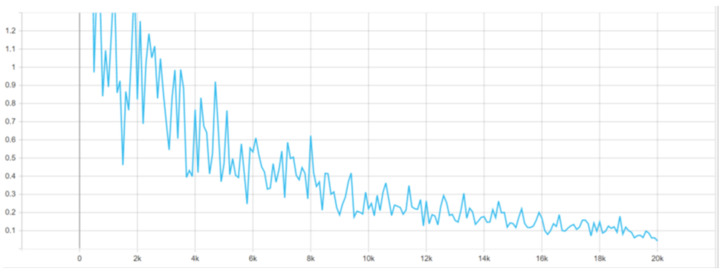
Loss function graph for overall loss, network retraining.

The evaluation results clearly indicate that training Faster R-CNN (Figure 5) on the set did not yield the best results. The presented metrics, however, cannot be accurately assessed without testing the network on pictures of microstructures from the training set. Analyzing this solution, we can see one of the reasons why the evaluation results turned out to be so low. The network completely incorrectly detects form IV objects, where a large part of them have been classified as form VI objects. Those that are classified correctly have very low classification confidence. In the case of form I, the network coped very well in a large number of cases, achieving high confidence in classification. However, the network worked best for objects of form VI, where it achieved 100% classification certainty in most cases. It was decided to solve the problem of poor detection of form IV by once again preparing annotations on the same dataset. It was found that, perhaps when creating the annotation, the wrong label was mistakenly given to one of the microstructure images, which disrupted the learning process. The possibility of overfitting was eliminated by trying to train the network again on the same annotations but this resulted in no visible changes (Figure 6). After repreparing the data, the network was retrained.

The network was taught for a shorter time, only 20,000 steps, taking into account its stabilization from the previous process. This time, the accuracy scores during training were also looked at, where it was concluded that, between 16,000 steps and 20,000 steps, the precision of the network did not change significantly. The final model exported was the one created from 18,000 steps. During training, the charts showed a faster stabilization of the loss function results as well as smaller jumps than for the previous one, which confirmed the belief that in the case of the previous annotation, there must have been a mistake that negatively affected the entire learning process. For form VI, some of the objects are omitted but these are isolated cases. Form IV is also detected correctly, where agglomerated isolates are caused by the occurrence of such cases in the training set. This confirms the theory of a bug in the annotations of the previous model. The detector performed the worst on form I, which had the greatest variety of shapes, but many of the desired forms were correctly detected. Nevertheless, the fact is that, due to their location and shape, form I objects detected with the help of a classic detector give a hardly readable result. Summarizing the results of the experiments presented in this subchapter, the image detection algorithm Faster R-CNN is able to cope with the detection of objects of forms I, IV and VI precipitates using the annotations generated by the solution presented in this paper. However, the results are not perfect, as the evaluation shows. After inspecting the presented examples, it can be concluded that form I objects pose the greatest challenge for the algorithm. Their detections are also very illegible, which results from the very shape and arrangement of objects in combination with the way of marking used by this type of solution.

#### 3.1.2. Mask R-CNN

With its use, a detector model was trained, which was trained for 110 epochs, each of which contained 50 steps. The appropriate number of classes was set according to the types of objects and the background was also taken into account. The network being the core of the solution was also changed from the Resnet101 network to the Resnet50 network, which was motivated by the impossibility of working with too large an architecture. Due to the long time needed to teach this solution, it was carried out in parts. One such part lasted from 5 to, most often, 15 epochs. The loss function information logged to Tensorboard was also not as well represented as in the previous solution, making it impossible to evaluate the model via loss function tracking. Fortunately, the model returned trained weights for each epoch that was performed, which allowed how the model copes with sample microstructure image to be verified at the end of each stage of training. After 110 epochs of 50 steps, learning is complete.

**Figure 7:**
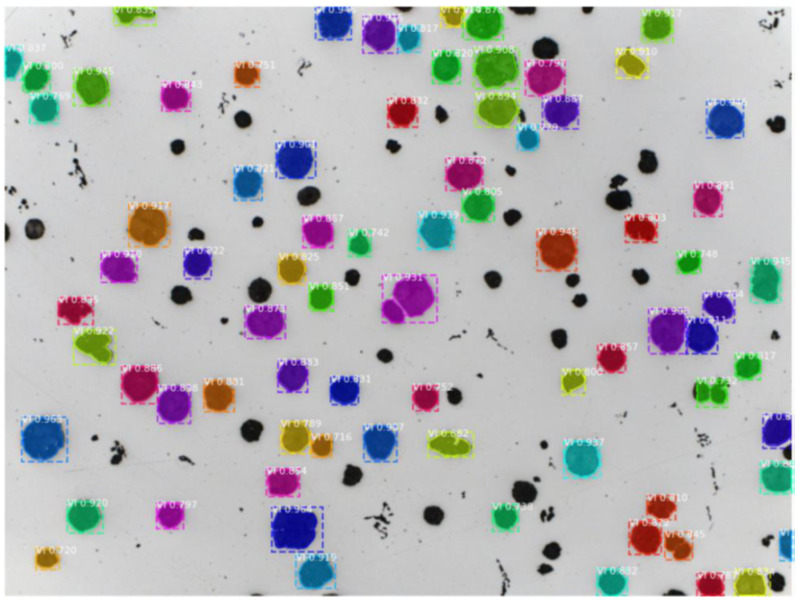
Operation of the Mask R-CNN detector on an image containing mostly form VI objects.

At the aforementioned stage of learning, the algorithm shows its effectiveness in operation on the prepared set. Both forms IV and VI are detected with high scores, the vast majority of which are over 0.8. Some form VI objects were not marked at all. This may be due to the lack of labels for form VI objects in the microstructure image where form IV objects were marked for the need for longer learning, which unfortunately was not allowed by hardware resources. Form I objects were not so well detected but their smaller silhouettes were correctly marked by the algorithm at this stage, additionally indicating a high confidence score, mostly exceeding 0.7. In addition, the algorithm was trained on the same set of annotations as the first Faster R-CNN training trial, meaning that despite having labels that disrupted the training of its predecessor, Mask R-CNN (Figure 7) correctly managed to learn the differences between form IV and VI objects. This may be due to the use of image segmentation, as the algorithm had more accurate information about the objects at its disposal.

## 4. Discussion

In the case of a classical detector using random forests or SVM as its classifier, it failed to achieve its intended goals. Classic classifiers in combination with the sliding window mechanism turned out to be not universal solutions because each type of detected object had to be approached individually and the detector parameters had to be changed manually to achieve the best results for them. Also, the need to eliminate one of the forms of the objects due to their too-diverse shape and layout meant that these were by far the worst-performing solutions. Another major disadvantage of these solutions was the way in which the data had to be prepared. Giving only examples of microstructure images with or without objects, treating them as separate microstructure images without the context of the rest of the image turned out to be an inefficient and difficult-to-configure method, especially in the case of manually marking the background. In addition, detections performed on a single image took an extremely long time. For effective detection, the sliding window had to have a very small step compared to the size of the image, similar to the coefficient for the Gaussian pyramid. This resulted in a very large number of necessary classifications, which, combined with average speeds per single classification, resulted in a very long waiting time. Of the two classifiers, random forests proved to be the more effective solution as they were better at learning to recognize full objects, while SVM often pointed to completely wrong areas of the image. Random forests also took much longer to classify than SVM.

Faster R-CNN and Mask R-CNN showed that both networks with the appropriate training set are able to cope with the task of detecting the tested objects. However, Mask R-CNN showed greater resistance to incorrect annotations, coping well with the use of a set where its predecessor failed. Faster R-CNN, after training it the second time, showed very good results for objects of form IV and VI, maintaining detection confidence measures above the threshold of 0.8 for almost all detected objects, while coping a little less well with form I, for which some objects were not correctly detected, and the results for correctly labeled ones were usually higher than 0.7. Mask R-CNN could not be trained as accurately as Faster R-CNN for technical reasons, but the results of the detections made on the test images indicated its high efficiency. In addition, Mask R-CNN, thanks to the use of segmentation, was able to indicate the exact contours of the detected object, which helped visualize the results. The method based on SVM and random forests showed one advantage over solutions using CNN. We are talking about the time it took to train the detector. Training a convolutional network takes a very long time. The conclusions from the conducted experiments are that solutions based on convolutional networks are better suited to the task of detection and classification of objects of forms I, IV and VI that make up the examined microstructures. The use of the created tool to create training and test sets resulted in the occurrence of a small number of incorrect determinations in the sets, which, in the case of solutions as sensitive to training data as the tested SVM, may cause a lot of false detections. It is also problematic to require a constant size of images from the training set because it causes problems with detecting objects of the same class with different shapes. The preferred solution for this type of problem should be networks using image segmentation because they are less sensitive to minor errors in training sets, and they also provide the possibility of more accurate analysis by marking the contours of the found instance and not just the frame around it.

## 5. Conclusions

The conclusions from the conducted experiments are that solutions based on convolutional networks are better suited to the task of detecting and classifying objects in the examined case for forms I, IV and VI, constituting the examined microstructures. The use of the created tool to create training and test sets resulted in a small number of incorrect markings in the sets, which, in the case of solutions as sensitive to training data as the tested SVM, may result in many incorrect detections. It is also problematic to require a constant size of photos from the training set because it causes problems with detecting objects of the same class with different shapes. The preferred solution for this type of problem should be networks using image segmentation because they are less sensitive to minor errors in training sets, and they also provide the possibility of more detailed analysis by marking the contours of the found instance and not just the frame around it.

The application created in this work made it possible to facilitate and speed up this process but also allowed us to check how the set created in this way would affect the training of detectors. However, it has been shown that the Faster R-CNN and Mask R-CNN algorithms, even on less accurate training data generated with the help of the tool, are able to learn to correctly detect the desired objects. It was also noticed that the classic image detection method, despite its ability to cope with detection, is too uncertain and difficult in the appropriate configuration to achieve sufficiently accurate results. In the case of convolutional networks, for the Faster R-CNN solution, an error in the annotation of some objects turned out to be so important that it disrupted the training process. This sensitivity was noticed after the correct operation of Mask R-CNN, trained on the same annotations, which showed its greater resistance to this type of error. The developed solution was tested in laboratory conditions SBŁ-KIT. 

## Figures and Tables

**Table 1:** Accuracy metric results for the SVM classifier obtained from the scikit-learn library.

	Precision	Recall
I	1.00	0.99
IV	0.98	0.91
VI	0.92	1.00
Background	1.00	0.99

**Table 2:** Random forest classifier accuracy metrics results from scikit-learn.

	Precision	Recall
I	1.00	1.00
IV	1.00	0.92
VI	0.93	1.00
Background	1.00	1.00

**Table 3:** Average precision metrics for 0.50 and 0.75 thresholds for a trained detector.

AP 0.5	AP 0.75
0.661	0.508

## Data Availability

Not applicable.
